# A Simplified Calculation Method of Heat Source Model for Induction Heating

**DOI:** 10.3390/ma12182938

**Published:** 2019-09-11

**Authors:** Hongbao Dong, Yao Zhao, Hua Yuan, Xiaocai Hu, Zhen Yang

**Affiliations:** 1 School of Naval Architecture and Ocean Engineering, Huazhong University of Science and Technology, Wuhan 430074, China; 2 Collaborative Innovation Center for Advanced Ship and Deep-Sea Exploration (CISSE), Shanghai 200240, China; 3 Hubei Key Laboratory of Naval Architecture and Ocean Engineering Hydrodynamics (HUST), Wuhan 430074, China; 4 Shanghai Waigaoqiao Shipbuilding Co., Ltd., Shanghai 200137, China

**Keywords:** induction heating, heat source model, shape parameters, Gaussian function

## Abstract

Line heating is used in forming the complex curve plates of ships, and this process is becoming integrated into automated tools. Induction heating equipment has become commonly used in automatic line heating. When applying automated equipment, it is necessary to calculate the relationship between the heating parameters and the temperature field. Numerical methods are primarily used to accomplish the calculations for induction heating. This computation process requires repeated iterations to obtain a stable heat generation rate. Once the heat generation rate changes significantly, a recalculation takes place. Due to the relative position of the coil and plate changes during heating, the grid needs to be frequently re-divided during computation, which dramatically increases the total computation time. In this paper, through an analysis of the computation process for induction heating, the root node that restricts the computation efficiency in the conventional electromagnetic-thermal computation process was found. A method that uses a Gaussian function to represent the heat flux was proposed to replace the electromagnetic computation. The heat flux is the input for calculating the temperature field, thus avoiding the calculation of the electromagnetic analysis during induction heating. Besides, an equivalence relationship for multi-coil was proposed in this paper. By comparing the results of the experiment and the numerical method, the proposed heat source model’s effectiveness was verified.

## 1. Introduction

Line heating is a typical process in the plate forming of ships. In the past, the operation was mostly manual and included the use of a flame heater. With advancements in research and the development of automated line heating equipment, flame heating by oxyacetylene combustion has shown its limitations because of the difficulty with temperature control. Induction heating is used extensively for the forming of plates because it is easily controlled and has high efficiency.

**Figure 1:**
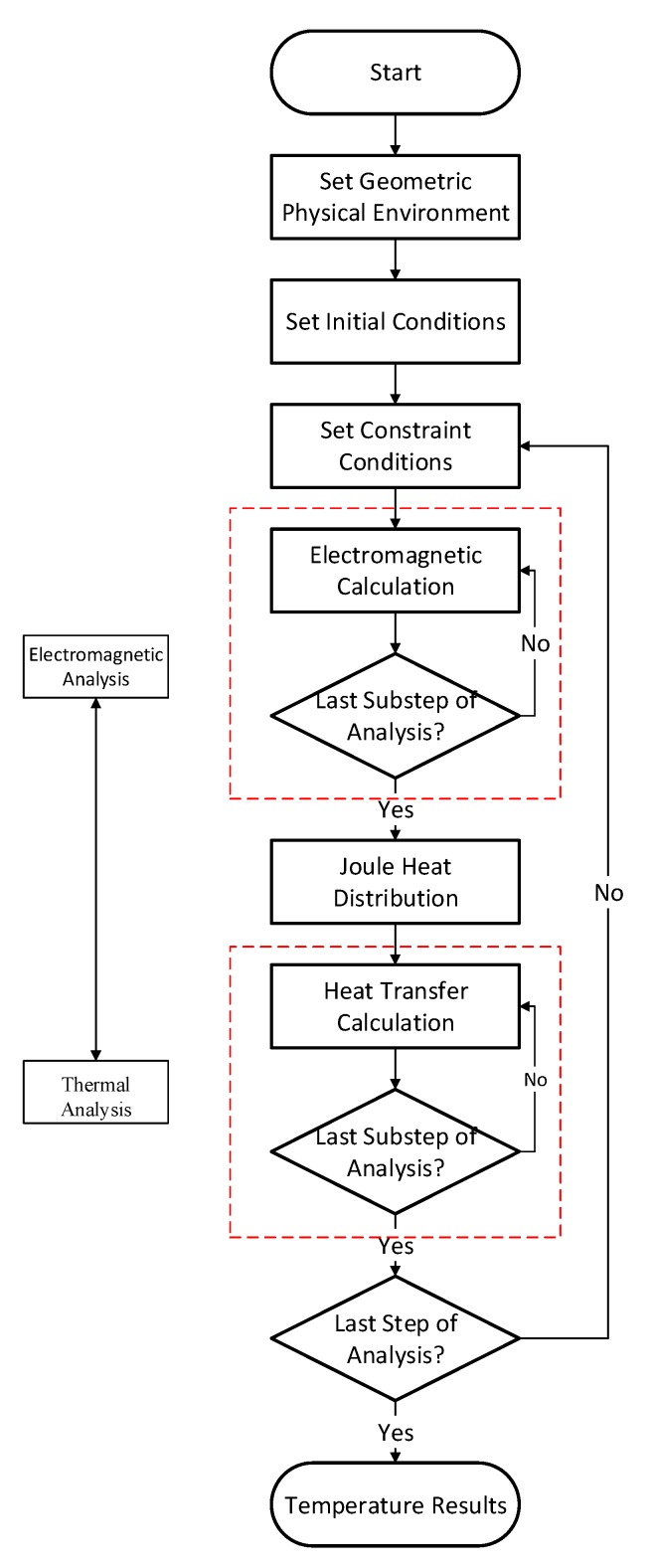
Electromagnetic-thermal computation process.

The principle of electromagnetic heating is to generate an eddy current in a workpiece, which is then heated by the Joule heat generated by the workpiece’s resistance. An induction heater includes a power source and an induction coil. The shape of the induction coil varies based on the object to be heated. The induction heating process includes electromagnetic induction and heat conduction. It is tough to obtain the temperature distribution of the workpiece during induction heating through a theoretical method and experiment-based methods are too costly. Therefore, numerical simulation has become an effective solution for obtaining the temperature fields of induction heating. A typical process for induction heating simulation is presented in Figure 1. First, time-harmonic electromagnetic analysis is performed with the designated initial conditions and constraint conditions under a given frequency and power. The Joule heat distribution of the induction coil is calculated when a stable heat generation rate is obtained. The temperature field of the workpiece can be calculated using the Joule heat distribution as the input of thermal analysis. The thermal analysis ends when a stable node temperature is obtained. If the set calculation time has not been reached, the induction coil is then moved to the next position. A new analysis is carried out when establishing the constraint equations about the coil-workpiece-air interface.

In the computation process mentioned above, it is necessary to calculate the electromagnetic field and the temperature field. The Joule heat obtained from the electromagnetic calculation is the input of the heat transfer calculation. It is necessary to frequently re-divide the grid based on the coil position when performing moving induction heating forming simulations, which increase the time of computations and decrease computation efficiency.

Some studies have investigated methods to improve computation efficiency. Monzel [1] used the neural network method to carry out preliminary research on the features of the inductive current distribution during transverse flux induction heating. He gave measures for reducing cable loss by changing each coil’s current to change the heat source distribution. The typical form of heat source density was given in the study, but the corresponding simplified numerical method was not mentioned. Bay [2] postulated that the temperature calculation relative to the electromagnetic calculation was a quasi-static process and proposed mathematical and numerical models for the coupled axisymmetric induction heating process. The model simplified the full-coupling process into a weak coupling process, used the eddy current obtained in the electromagnetic calculation as the heat generation rate in the temperature calculation, and presented a preliminary calculation method for the heat generation rate, but the expression for the heat source model was not given. Luo [3,4,5,6] carried out finite element calculations on the temperature field and the deformation field during high-frequency induction heating. He compared the relevant results with the experimental results, which avoided the electromagnetic coupling calculation. He gave the corresponding heat source model, but the calculation method for the heat source model and the corresponding relationship of various parameters in the model with the actual coil were not discussed. Liu [7] presented a numerical method for the temperature field of induction heating and studied the law of temperature distribution. When handling the electromagnetic-thermal coupling problem, a heat source model based on an empirical formula was used, but the calculation process for the model was not given. Hu [8] proposed the use of an equivalent heat source to replace the electromagnetic coupling calculation in induction heating and carried out experimental verification of the proposed heat source model, but did not give an explicit expression for the heat source model. Zhang [9] analyzed the similarities and differences of induction heating and flame heating in the line heating of ship plates and thought that induction heating was feasible in heat forming. When handling the induction heating process, he provided a heat source model for induction heating and used it as the initial input for the thermal distribution calculation. Compared with the experimental results, the errors of the calculation results satisfied practical engineering requirements. However, the model was simplified and the meaning of various model parameters and the corresponding numerical methods were not explained. Bae [10] proposed a two-dimensional circular heat input model to simulate the induction heating process and obtained satisfactory results, but the expression of the heat source model was not taken into account. Jeong [11] simplified coupled induction heating into the electromagnetic calculations and thermal calculations. He used statistical methods to describe the correlation between deformation and the input parameters, but the expression of the heat source model was not described in the study. Bai [12] used stepwise analysis to carry out coupling analysis within a typical time to obtain the state of induction heat distribution. He used the distribution as the moving heat source for a subsequent calculation, but the expression for the heat source was not given. Kubota [13] used a three-dimensional coupling and heat source model to perform calculations on the induction heating process and found that the results were consistent with measured results. The heat source model was obtained based on the obtained temperature field. Yang [14] proposed a heat source model based on the characteristics of the induction heating eddy current and used the finite element method (FEM) and relevant tests to verify the effectiveness of the model. The proposed heat source model was based on empirical methods and only targeted a particular coil form without giving the expression form of the corresponding heat source for other coil forms. Through theoretical analysis, Li [15] studied the analytical solution for obtaining the heat generation rate in the semi-infinite space during induction heating and discussed the effects of relevant parameters on the heat generation rate. To compare the difference in residual stress in a plate after single heating and double heating, Aung [16] proposed numerical methods based on a surface heat source and a body heat source. The effectiveness of the heat source models was verified through the experiment. Aung provided the expression form for the heat source model but did not discuss its calculation. Riccio [17] used different numerical models to study the bonding of carbon fiber reinforced polymer components with induction heating. ABAQUS was used to carry out electromagnetic analysis to obtain the energy loss caused by the Joule effect. He provided the finite element calculation model but not the expression for the Joule heat of the coil. In other studies related to the induction heating process and induction coil design [18,19,20], the full-coupling numerical method was also used for the induction heating calculations. Although the results were satisfactory, the time cost was high and the applicability was low for coils of different shapes and sizes or with different processing conditions. Zhang [21] replaced the electromagnetic-thermal coupling calculations with a heat source model corresponding to a discrete form and proposed a simplified calculation method for the high-frequency induction heat source. The computation efficiency for the calculation of a certain specific induction heating parameter was greatly improved, but any change in the induction heating parameters necessitated repeating the entire calculation.

The induction heating calculation, especially the direct calculation of the moving induction heating process, results in a long computation time due to the need to continuously change the grid. However, the use of the heat flux to replace the coil can achieve high precision and reduce the computation time. Currently, most studies focus on empirical methods, and there are comparatively few studies that calculate the heat source and investigate the effect of the coil on the model. Based on these, the analytical method was employed in this study to calculate the heat source model for the induction coil. The effects of coil shapes on the heat source were examined. In addition, related experiments were conducted to verify the obtained heat source model for single-coil and multi-coil.

## 2. Simplification of Heat Source

### 2.1. Finite Element Calculation

In the induction heating process, the input is alternating current. The alternating current generates an alternating magnetic field in the coil which generates an induced electric field in the workpiece, which in turn generates heat in the workpiece. The electromagnetic field in the induction heating process is given by Maxwell’s equations:

(1) $$\nabla \times \vec{H}=\vec{J}+\frac{\partial \vec{D}}{\partial t}$$

(2) $$\nabla \times \vec{E}=-\frac{\partial \vec{B}}{\partial t}$$

(3) $$\nabla \cdot \vec{B}=0$$

(4) $$\nabla \cdot \vec{D}=\rho$$

where $\vec{H}$ is the magnetic field strength, $\vec{J}$ is the conduction current density, $-\frac{\partial \vec{D}}{\partial t}$ is the displacement current density, t represents time, $\vec{E}$ is the electric field, $\vec{B}$ is the magnetic field, $\vec{D}$ is the electric flux density, and ρ is the volume charge density.

The corresponding auxiliary equations are:

(5) $$\vec{D}=\varepsilon \vec{E}$$

(6) $$\vec{B}=\mu \vec{H}$$

(7) $$\vec{J}=\sigma \vec{E}$$

where ε represents the dielectric constant, μ is the magnetic permeability, and σ is the electrical conductivity.

The eddy current density distribution in the workpiece can be obtained by solving the aforementioned equations and the distribution of the heat generation rate can be obtained using Joule’s law:

(8) $$q=\frac{{\left|\vec{J}\right|}^{2}}{\sigma }$$

where q represents the heat generation rate.

The heat generation rate is used as the input in the thermal conduction equation for calculating the thermal distribution, and the temperature field of the plate is obtained:

(9) $$\rho c\frac{\partial T}{\partial t}=\frac{\partial }{\partial x}\left(\lambda \frac{\partial T}{\partial x}\right)+\frac{\partial }{\partial y}\left(\lambda \frac{\partial T}{\partial y}\right)+\frac{\partial }{\partial z}\left(\lambda \frac{\partial T}{\partial z}\right)+q$$

where ρ is the density, c is the specific heat, T represents the temperature, and λ represents the thermal conductivity.

The above procedure is the detailed process for the calculation of the induction heating. It is necessary to continuously update the corresponding parameters with temperature changes during the calculation as the workpiece’s electromagnetic and thermal property parameters are related to the temperature, which leads to an extremely time-consuming computation process. When the coil and the workpiece have relative movement, the computation process, as mentioned earlier, must be repeated to obtain the temperature field during movement.

### 2.2. Simplification of Heat Source Model

**Figure 2:**
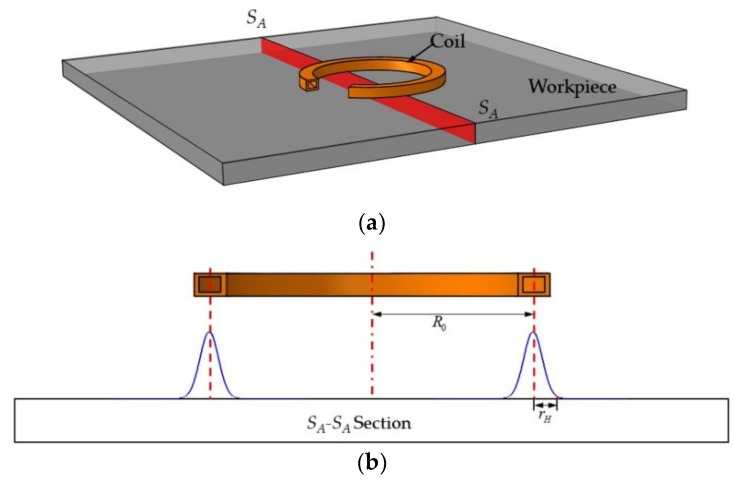
Diagrams of computation model and heat source distribution: (**a**) Computation model and (**b**) Gaussian distribution of the heat source.

The key to temperature calculation is in obtaining the distribution of the heat generation rate q. Due to the skin effect, the heat generated by the induction is concentrated on the surface of the workpiece so the surface heat flux can be used as the input during induction heating. The heat flux model used in this paper is shown in Figure 2. The cumbersome electromagnetic calculation is unnecessary once the heat flux model is used to represent the coil.

The heat flux along the surface of the workpiece in the radial direction of a single coil was assumed to follow a Gaussian function:

(10) $$q\left(r\right)=q_{m}e^{-{\left(\frac{r-R_{0}}{r_{H}}\right)}^{2}}$$

where q(r) represents the heat flux at a distance r from the center of the heat source, r is the coordinate along the radial direction of the coil, $q_{m}$ is the maximum heat flux, $R_{0}$ represents the radius of the heat source, and $r_{H}$ represents the effective radius of the heat flux.

The total power on the workpiece is equal to the effective power Q of the induction coil, that is:

(11) $$Q=\int_{0}^{2\pi }R_{0}d\theta \int_{-\infty }^{+\infty }q\left(r\right)dr=2\pi \sqrt{\pi }q_{m}R_{0}r_{H}$$

which leads to:

(12) $$q_{m}=\frac{Q}{2\pi \sqrt{\pi }R_{0}r_{H}}$$

Thus:

(13) $$q\left(r\right)=\frac{Q}{2\pi \sqrt{\pi }R_{0}r_{H}}e^{-{\left(\frac{r-R_{0}}{r_{H}}\right)}^{2}}$$

Equation (13) is the heat flux corresponding to the coil. The temperature field of a workpiece can be directly calculated after the heat flux is substituted into Equation (9). Since the Joule heat distribution is obtained directly from Equation (13) to represent the induction coil, the electromagnetic calculation in Figure 1 is unnecessary, and the heat transfer is directly calculated. Moreover, grid redivision during coil movement is unnecessary, resulting in fast computation.

## 3. Verifications of Heat Source Model

### 3.1. Experimental Verification for Single Coil

**Figure 3:**
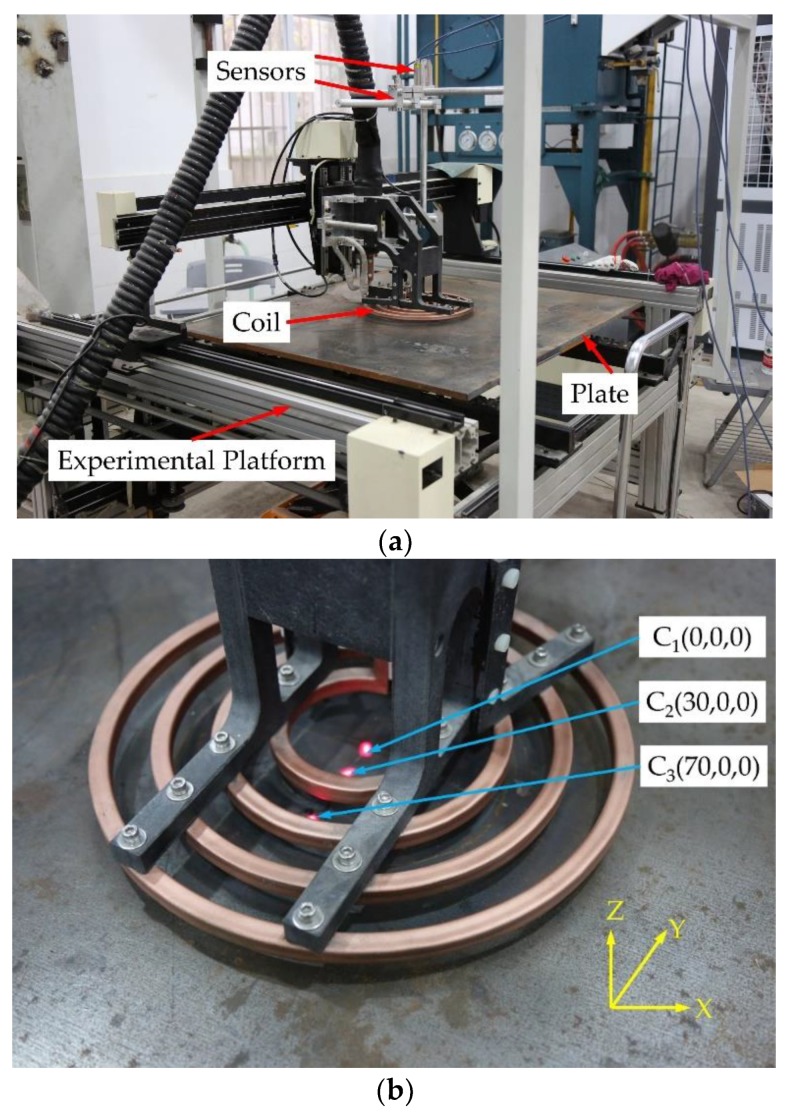
Schematic diagram of the induction heating experiment: (**a**) Experimental platform for induction heating and (**b**) Positions of the temperature measurement points.

The heat flux model of a coil during induction heating has been previously stated, and a static induction heating experiment was conducted on a three-axis motion platform to verify the effectiveness of the model. The platform had degrees of freedom in the three directions of X-Y-Z with a positional precision of 1 mm and a velocity precision of 1 mm/s. The schematic diagram of the induction heating experiment is shown in Figure 3.

The experimental plate was low carbon steel and the material properties were similar to those in the Reference [22], as listed in Table 1 and Table 2. The size of the plate was 1000 mm × 800 mm × 16 mm.

**Figure 4:**
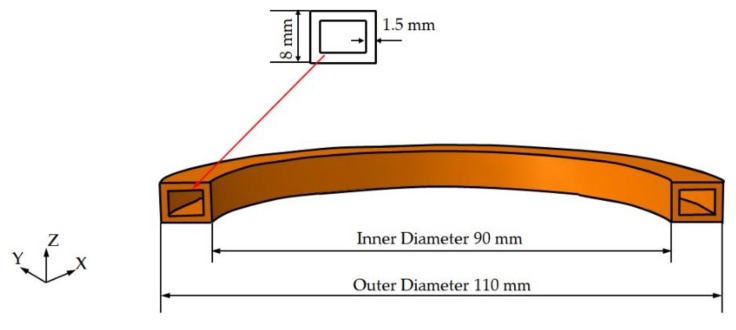
Schematic diagram of the single coil.

A ring-type coil was used in the experiment, as shown in Figure 3. The induction coil was copper with an inner diameter of 90 mm and an outer diameter of 110 mm. The cross-section of the coil was a rectangle with a size of 10 mm × 8 mm and a wall thickness of 1.5 mm. The schematic diagram of the single coil is illustrated in Figure 4.

The frequency of induction heating during the experiment was 12 kHz and the input power was 7.5 kW. The distance between the coil and the surface of the plate was 5 mm, which remained unchanged during heating. The heating time was 120 s. The induction heating efficiency was assumed to be 80%. An infrared temperature sensor (with a measurement range of 250 °C–1450 °C) was used to measure the temperature history of the plate. The distances between the sensors and the center of the coil were 0 mm, 30 mm, and 70 mm. A schematic diagram of the temperature measurement points is shown in Figure 3b.

### 3.2. FEM Calculation and Comparison

Following experimental conditions, the heat flux function of the coil during heating can be calculated based on the input parameters from Equation (13):

(14) $$q\left(r\right)=5.39\times 10^{5}\times e^{-{\left(\frac{r-0.05}{0.02}\right)}^{2}}$$

**Figure 5:**
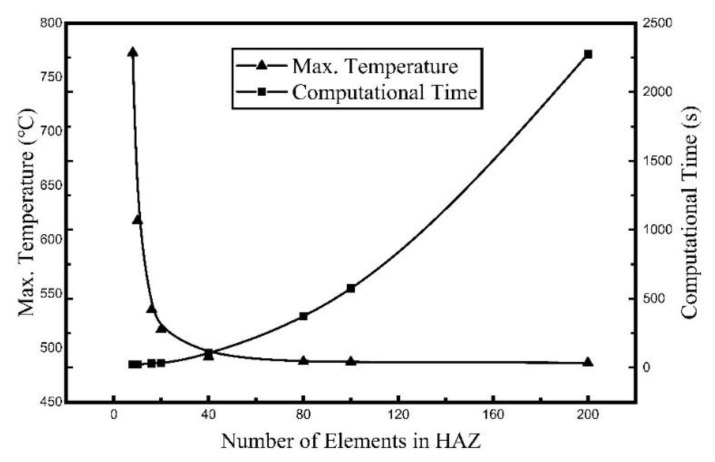
Relationship between maximum temperature and computational time and number of elements in the heat affected zone (HAZ).

The heat flux obtained was used as the input of the heat transfer calculation to obtain the temperature field of the plate. ABAQUS was used to carry out the heat transfer calculation. The plate size was 1000 mm × 800 mm × 16 mm. The element type of the plate is DC3D8 for heat transfer simulation. The finite element model under different grid sizes was established to investigate the effect of the element size. The correlation between the maximum temperature and calculation time and the number of grids in the heat affected zone (HAZ) was compared. The width of the HAZ was 400 mm. The grids of the length direction and the width direction were the same sizes, and the thickness direction was unified into four grids. All calculations were performed on the same computer: Windows 10, 64 bits, four cores with 4.2 GHz and 8 GB of RAM. The relationship between the maximum temperature of the plate and calculation time and the number of elements in HAZ is given in Figure 5.

**Figure 6:**
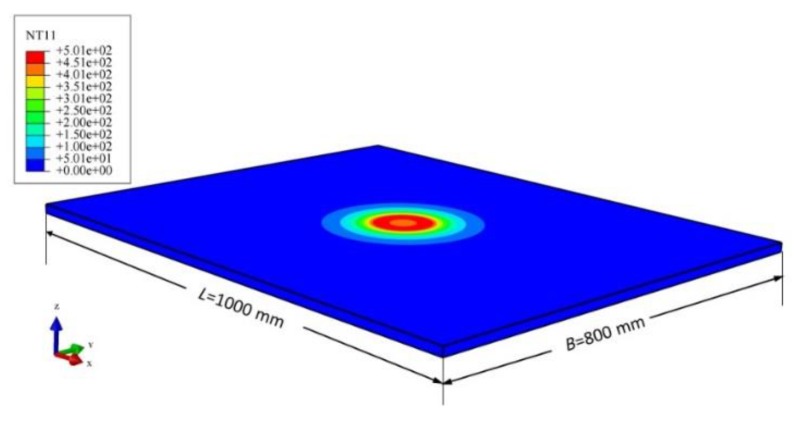
The finite element result of the heat transfer calculation.

As seen from Figure 5 that the maximum temperature gradually decreases and reaches a stable value as the number of grids increases to 80 (which means the element size is 5 mm). However, the computing time increases sharply when the number of grids is over 100 (which means the element size is 4 mm). We can conclude that the usage of an element of size equal to 5 mm is appropriate in reasonable computational time without losing appreciable accuracy. A variable grid was used for element division. The grids in the HAZ were 5 mm × 4 mm × 4 mm and the elements far from the heating zone were 50 mm × 20 mm × 8 mm. The total number of elements was 68,600 and the number of grids was 86,399. The finite element result is given in Figure 6.

**Figure 7:**
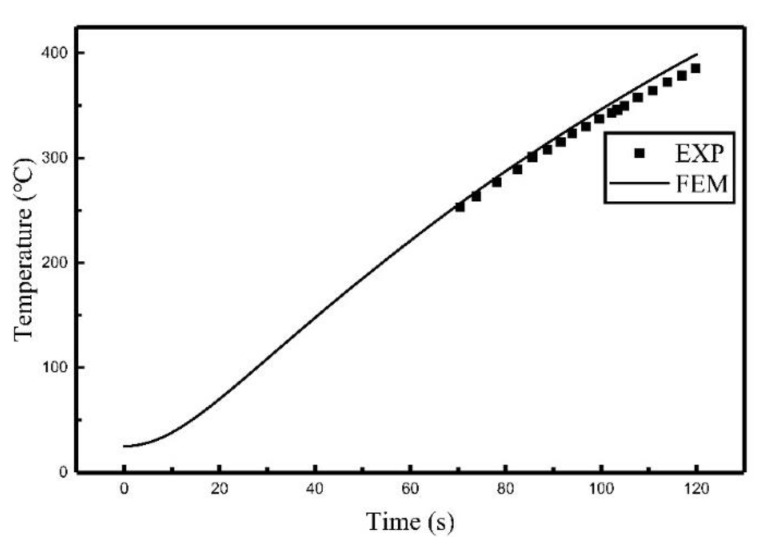
Comparison of the temperature histories of C_1_.

**Figure 8:**
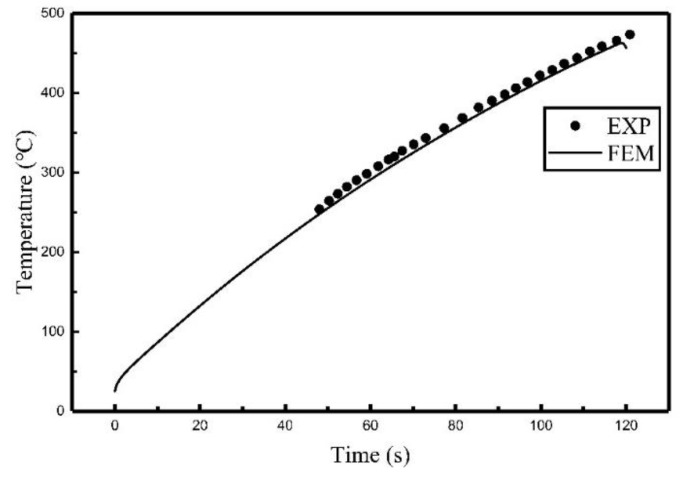
Comparison of the temperature histories of C_2_.

**Figure 9:**
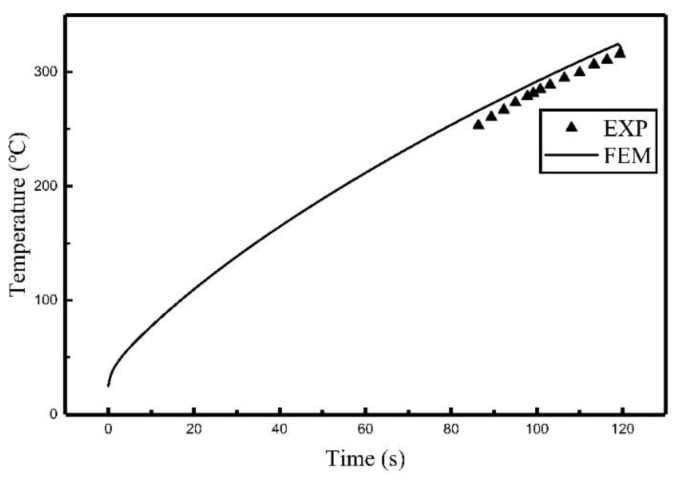
Comparison of the temperature histories of C_3_.

Comparisons of the experiment results and finite element calculation using the heat source model are shown in Figure 7, Figure 8 and Figure 9, the relative locations of C_1_, C_2_, and C_3_ are shown in Figure 3.

It can be seen from Figure 7, Figure 8 and Figure 9 that the temperature histories obtained using the heat source model for calculation are in agreement with the experiment results. Table 3 summarized the relative error between simulated results and experimental results. It can be seen that the maximum relative error was 5.42% and the average relative error was 3.64%. The comparison indicates that the temperature field of induction heating can be accurately simulated using a heat source model.

**Figure 10:**
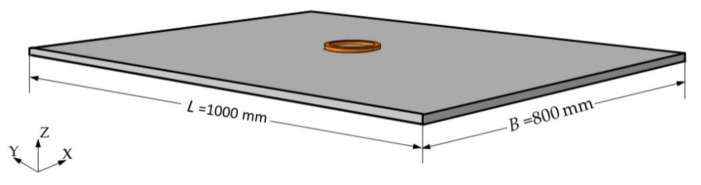
Coupling calculation model.

The computation efficiency using the heat source model was compared with that using the full-coupling method. COMSOL Multiphysics was used to carry out the coupling calculations. The maximum element size of the model was 1 mm. The total number of elements was 34,274. The coupling model is shown in Figure 10.

**Figure 11:**
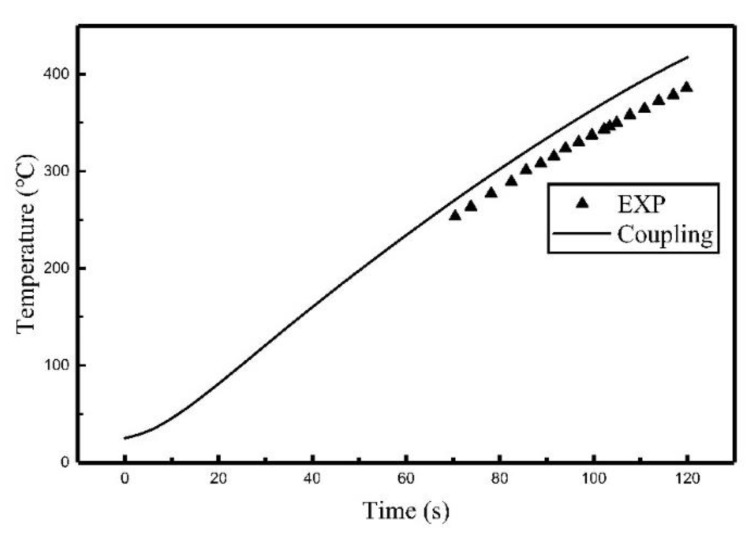
Comparison of the temperature histories of C_1_ between the coupling model and the experiment.

**Figure 12:**
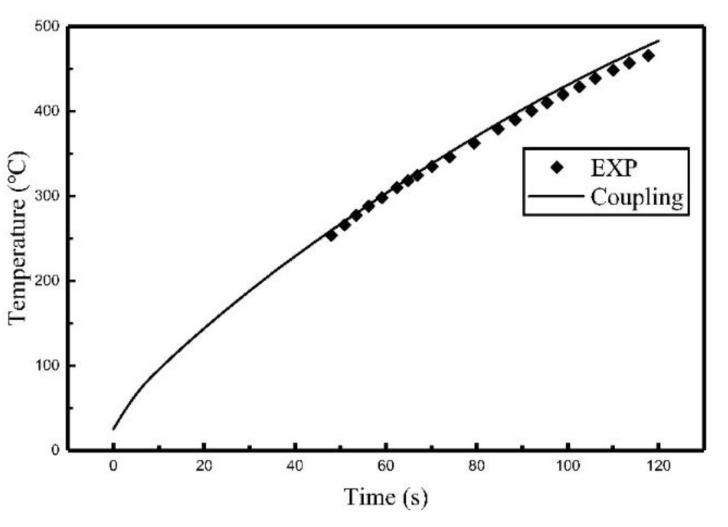
Comparison of the temperature histories of C_2_ between the coupling model and the experiment.

**Figure 13:**
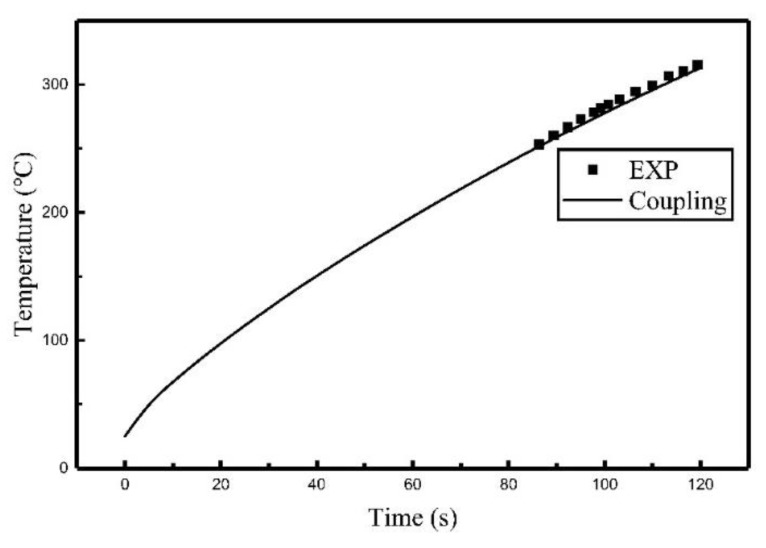
Comparison of the temperature histories of C_3_ between the coupling model and the experiment.

The comparisons between the coupling model and the experiment are shown in Figure 11, Figure 12 and Figure 13. The comparison of relative error between coupling model and the experiment are listed in Table 4.

As can be seen from Figure 11, Figure 12 and Figure 13, the temperature histories obtained by the coupling model are in agreement with the experiment results. The relative error between the simulated results and experimental results, listed in Table 4, showed that the average relative error was 7.55% at most, which means the chosen simulation method was correct for calculating induction heating.

Table 5 shows the number of degrees and computing time between the coupling model and the heat source model. The number of degrees of freedom and the computation time during coupling computing was higher than the heat source model. It can be considered that the heat source model has sufficient accuracy and efficiency for induction heating calculation.

### 3.3. Application to Multi-Coil

It can be seen from Equation (13) that the Gaussian function that represents the heat source has a relationship with the project area of the coil. To further verify the effectiveness of the proposed heat source model, the previously described coil, shown in Figure 3b, was used to conduct a multi-coil experiment.

**Figure 14:**
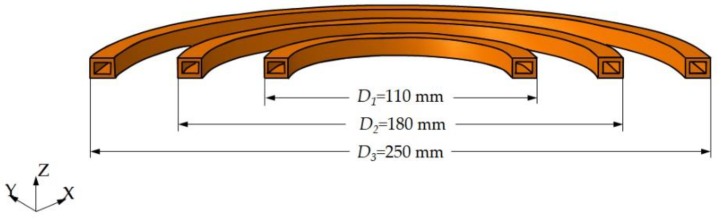
Schematic diagram of sizes of multiple coils.

A static induction heating experiment of multi-coil was conducted on the previously described experimental platform. The schematic diagram of the multi-coil is shown in Figure 14. The heating power was 25 kW and the heating time was 100 s. The bottom of the coil was 5 mm from the surface of the plate. The size of the plate and positions of the temperature measurement points were the same as those in the previously described experiment. The experimental results were compared with the finite element results calculated using the heat source model.

During the experiment, the heat flux was more diffused along the radial direction of the coil and its scope of influence had become larger. Therefore, the action radius of the heat flux was assumed to be$\;r_{H}=0.04$, the position of the heat flux center was at $R_{0}=0.085$ in the middle coil, and the heat flux was given by:

(15) $$q\left(r\right)=5.28\times 10^{5}\times e^{-{\left(\frac{r-0.085}{0.04}\right)}^{2}}$$

**Figure 15:**
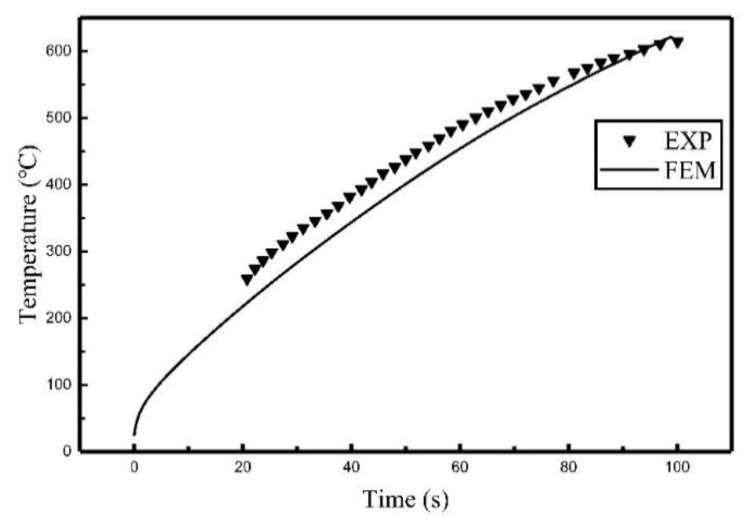
The temperature histories of C_3_ during the multi-coil experiment and simulation.

**Figure 16:**
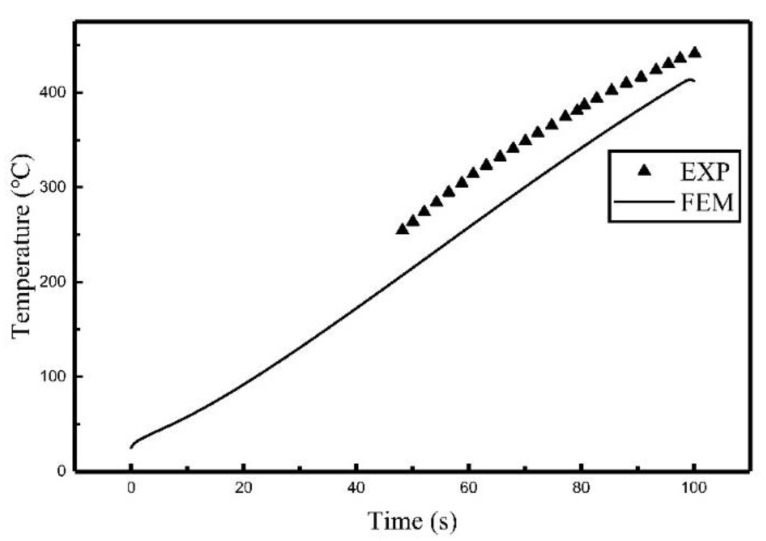
The temperature histories of C_2_ during the multi-coil experiment and simulation.

Comparisons of the simulation results and the experiment results are shown in Figure 15 and Figure 16. The error comparison between simulation results and experimental results are listed in Table 6.

It can be seen from Figure 15 and Figure 16 and Table 6 that the results of the heat source model are in agreement with the results obtained in the experiment in terms of the trend with a maximum relative error of 18.6%. It means that the proposed heat source model is also applicable for use with multi-coil induction heating, which expands the application of the heat source model.

## 4. Conclusions

In this paper, the heat flux of the coil during induction heating was studied. The expression of the heat flux was obtained and the accuracy was verified through calculation and experiment. The following conclusions were drawn:

1. The heat flux during induction heating can be simplified into a Gaussian function, and the values of the Gaussian function are related to the size of the induction coil and the heating conditions.

2. The heat source model can be used to calculate the temperature distribution of induction heating. The proposed model can improve computation efficiency while ensuring precision. Fast computation of moving induction heating can be achieved using this model.

3. The proposed heat source can also be used in multi-coil induction heating, as the goodness of the finite element results and the experimental result is within an acceptable range.

## Figures and Tables

**Table 1:** Thermal parameters of low carbon steel.

Temperature (°C)	Density (kg/m3)	Specific Heat (J/(kg·°C))	Heat Conductivity Coefficient (W/(m·°C))
0	7842	450.36	66.97
50	-	464.6	65.21
200	7822	498.1	57.38
250	-	502.26	54.91
300	-	514.82	53
400	7802	537.42	47.92
450	-	623.64	45.83
500	-	707.35	43.53
600	7782	812	39.3
650	-	904.07	36.37
700	-	967.69	34.74
800	7761	1026.32	31.02

**Table 2:** Mechanical parameters of low carbon steel.

Temperature (°C)	Young‘s Modulus (GPa)	Poisson Ratio	Heat Expansion Coefficient (1/°C)	Yield Strength (MPa)
0	206	0.267	1.20 × 10^−5^	235
50	196	0.29	1.25 × 10^−5^	-
200	196	0.322	1.40 × 10^−5^	163
250	186	0.296	1.43 × 10^−5^	-
300	186	0.262	1.47 × 10^−5^	-
400	166	0.24	1.54 × 10^−5^	130
450	157	0.229	1.57 × 10^−5^	-
500	157	0.223	1.59 × 10^−5^	-
600	135	0.223	1.64 × 10^−5^	119
650	117	0.223	1.66 × 10^−5^	-
700	112	0.223	1.67 × 10^−5^	-
800	113	0.223	1.69 × 10^−5^	109

**Table 3:** Comparison between simulated results and experimental results.

Sensor	Maximum Relative Error	Average Relative Error	Minimum Relative Error
C_1_	3.55%	2.43%	1.15%
C_2_	3.84%	2.42%	1.16%
C_3_	5.42%	3.64%	2.46%

**Table 4:** Comparison of coupling model and experiment.

Sensor	Maximum Relative Error	Average Relative Error	Minimum Relative Error
C_1_	8.65%	7.55%	6.38%
C_2_	2.71%	1.68%	0.50%
C_3_	1.61%	1.17%	0.27%

**Table 5:** Comparison of computation efficiency.

	Number of Degrees of Freedom	Computation Time/Min
Full-coupling model	230,917	6.7
Heat source model	86,399	3.7

**Table 6:** Comparison of simulation and experiment.

Sensor	Maximum Relative Error	Average Relative Error	Minimum Relative Error
C_2_	18.63%	12.72%	4.82%
C_3_	15.42%	7.65%	0.15%
